# The role of corneal biomechanics in visual field progression of primary open-angle glaucoma with ocular normotension or hypertension: a prospective longitude study

**DOI:** 10.3389/fbioe.2023.1174419

**Published:** 2023-05-10

**Authors:** Yahui Wei, Yu Cai, Chenying Bao, Yanfei Zhu, Yingzi Pan

**Affiliations:** Department of Ophthalmology, Peking University First Hospital, Beijing, China

**Keywords:** open-angle glaucoma, visual field progression, corneal biomechanics, normal tension glaucoma, CorVis ST

## Abstract

**Introduction:** To analyze effects of dynamic corneal response parameters (DCRs) on visual field (VF) progression in normal-tension glaucoma (NTG) and hypertension glaucoma (HTG).

**Methods:** This was a prospective cohort study. This study included 57 subjects with NTG and 54 with HTG, followed up for 4 years. The subjects were divided into progressive and nonprogressive groups according to VF progression. DCRs were evaluated by corneal visualization Scheimpflug technology. General linear models (GLMs) were used to compare DCRs between two groups, adjusting for age, axial length (AL), mean deviation (MD), etc. VF progression risk factors were evaluated by logistic regression and receiver operating characteristic (ROC) curves.

**Results:** For NTG, first applanation deflection area (A1Area) was increased in progressive group and constituted an independent risk factor for VF progression. ROC curve of A1Area combined with other relevant factors (age, AL, MD, etc.) for NTG progression had an area under curve (AUC) of 0.813, similar to the ROC curve with A1area alone (AUC = 0.751, *p* = 0.232). ROC curve with MD had an AUC of 0.638, lower than A1Area-combined ROC curve (*p* = 0.036). There was no significant difference in DCRs between the two groups in HTG.

**Conclusion:** Corneas in progressive NTG group were more deformable than nonprogressive group. A1Area may be an independent risk factor for NTG progression. It suggested that the eyes with more deformable corneas may also be less tolerant to pressure and accelerate VF progression. VF progression in HTG group was not related to DCRs. Its specific mechanism needs further studies.

## Introduction

Glaucoma is a leading cause of irreversible visual impairment and blindness worldwide (Tham YC et al., 2014). It is defined as a progressive optic neuropathy with characteristic changes in the optic nerve head (ONH) and corresponding visual field (VF) defects. Although intraocular pressure (IOP) is the most significant risk factor for glaucoma development and progression (Kass et al., 2002; Leske et al., 2007), reducing IOP to normal or even reducing it by 45% was still not enough to control VF progression clinically in some primary open-angle glaucoma (POAG) patients (Leske et al., 2004). Another group (Susanna et al., 2019) found in a study of 334 glaucoma patients with an average follow-up time of 4.3 years that approximately a quarter of patients with seemingly well-controlled IOP (IOP ≤ 18 mmHg) still had VF progression during follow-up. Although high IOP is considered the most important risk factor for glaucomatous optic nerve (GON) damage, there is still confusion about which other factors, such as hypoperfusion or parapapillary vascularity, are also risk factors for glaucomatous VF progression.

Recently, our study and several other studies showed that the corneas in normal-tension glaucoma (NTG) were softer and easier to deform than that in hypertension glaucoma (HTG) or the normal physiological state (Wei et al., 2021; Wu et al., 2022; Xu et al., 2022). Another study by our group (Li et al., 2017) also found that the corneas were more deformable in the worse eyes of asymmetric NTG patients. This finding provided evidence for the NTG pathophysiology hypothesis that lower tolerance for normal IOP of softer eyeballs causes mechanical damage. The above results have drawn scholars’ attention to the role of corneal biomechanics in glaucomatous VF progression.

Some studies have found that the corneal biomechanical characteristics (CBCs) of glaucoma patients were related to VF progression (Li et al., 2018; Jung et al., 2019). Most studies believed that the more corneal deformation ability of the eyeball, the faster the visual field progressed. CBCs may also provide some explanations for the VF progression of some glaucoma patients with seemingly well-controlled IOP. Jung et al. (2019) found that the deformation amplitude (DA) was increased in eyes with progressive POAG, that is, the deformability of the cornea was increased. A prior study of our group (Li et al., 2018) found that the time at the first applanation (A1T) in the progressive NTG group was reduced, while the DA was increased; that is, the progressive eyes were more easily deformed. Recently, the results of a study (Qassim et al., 2021) was contradictory to the conclusions of previous studies. They followed up 228 glaucoma suspect eyes (with glaucomatous optic nerve defects but without visual field damage) for 4.2 years and found that thin (smaller central cornea thickness, CCT) and hard (greater stiffness parameter at the first applanation, SP-A1) corneas were more likely to progress. The contradiction may be due to the following reasons: When comparing the corneal biomechanical index SP-A1, the author merely corrected for CCT and did not correct for age or axial length (AL), which may affect the results to some extent (Liu et al., 2021; Chen et al., 2022). Second, IOP was not used as one of the criteria when the participants were enrolled; thus, it was impossible to distinguish between NTG and HTG in the study, which may have an impact on the results. The relationship between corneal biomechanics and glaucoma progression still needs further study.

In this paper, POAG was divided into NTG and HTG according to 24-h Goldmann applanation tonometry (GAT) measurement when firstly diagnosed, and these subjects were analyzed separately. The purpose of this study is to explore the role of CBCs in VF progression among NTG and HTG patients with well-controlled IOP, to probe the corneal biomechanical risk factors for VF progression, and to provide auxiliary evidence for clinical evaluation of POAG VF progression.

## Methods

### Subjects

This was a prospective cohort study. The patients were recruited consecutively from the Glaucoma Department of Ophthalmology at Peking University First Hospital diagnosed with POAG from October 2018 to January 2019. The institutional review board approved the study protocol, and the study was conducted in full accordance with the tenets of the Declaration of Helsinki. Informed consent was obtained from all volunteers before the study commenced.

POAG was defined as a glaucomatous optic disc (cup-to-disc ratio greater than 0.6, asymmetry of the cup-to-disc ratio ≥ 0.2 between eyes and the presence of local or diffuse retinal nerve fiber layer defects or neuroretinal rim defects in absence of any other abnormalities that could explain such findings) and/or with a corresponding glaucomatous VF defect with an open angle, with other secondary factors excluded. Patients were assigned to the HTG group or the NTG group based on 24-h IOP measured by GAT when first diagnosed.

The inclusion criteria for POAG patients were as follows: age over 40 years, best-corrected visual acuity (BCVA) of 20/40 or better, and astigmatism less than 3.0 diopters. Patients with any of the following criteria were excluded: corneal scarring, any trauma or a history of previous ocular surgery, inflammatory eye disease, and systemic disease conditions with a known or anticipated effect on dynamic corneal response parameter (DCR) measurement (including diabetes mellitus).

All subjects underwent a thorough ophthalmic evaluation, including slit-lamp biomicroscopy, fundus examination, GAT measurement and gonioscopy. All subjects underwent automated perimetry using a Humphrey Field Analyzer II (Carl Zeiss Meditec, Jena, Germany) with a full threshold 24–2 SITA standard program. Central corneal thickness (CCT) was measured with a Pentacam (Oculus Optikgeräte GmbH, Wetzlar, Germany). AL was measured using an IOL-Master 500 (Carl Zeiss Meditec, Jena, Germany). The duration of prostaglandin (PG) treatment was recorded in all POAG patients.

If both eyes of a POAG patient met the inclusion criteria, the eye with more severe glaucoma [defined as a lower mean deviation (MD) value] was included in the analysis.

### Corneal visualization scheimpflug technology measurements

All measurements obtained with the Corneal visualization Scheimpflug technology (CST) were taken by the same experienced technicians and captured automatically to minimize operator dependence. Only CST examinations with a quality score of “OK” were included in the analysis.

The DCRs evaluated in this study were deflection length, amplitude, area and time at the first and second applanation (Vinciguerra et al., 2016; Lopes et al., 2017; Vinciguerra et al., 2017). Briefly, a faster and larger deformation at the first applanation (A1), as well as a slower and smaller deformation at the second applanation (A2), indicated a more deformable cornea. That is, higher deflection amplitude (Amp), deflection length (Length), and deflection area (Area) and shorter time (T) at A1 and opposite parameters at A2 indicated a softer cornea.

### Glaucoma follow-up

Follow-up was performed every 3–4 months for 4 years in total. Routine ophthalmologic examination, GAT, VF, stereo fundus photography, and spectral-domain optical coherence tomography (RT-Vue 100, Optovue, Fremont, CA) were performed. NTG and HTG populations with target IOP were included in the final analysis. Target IOP was defined as follows (Group, 1998; Leske et al., 2004; Prum et al., 2016): ① NTG: 24-h mean IOP decreased by at least 30% compared with baseline 24-h mean IOP before treatment; ② HTG: IOP ≤ 21 mmHg, and the average 24-h IOP decreased by at least 20% from the baseline 24-h IOP before treatment. The subjects were divided into the progressive and nonprogressive groups according to their Advanced Glaucoma Intervention Study (AGIS) scores (Group, 1994). Visual field progression was defined as an increase of 4 or more points in the AGIS score compared to baseline on each of the 3 consecutive follow-up visual field examinations (Group, 1994).

### Statistical analysis

Statistical analyses were performed using SPSS software (V.18, IBM), Stata 15.1 (StataCorp LLC, TX, United States) and MedCalc 15.2.2 (MedCalc Software bvba, Ostend, Belgium). Categorical variables were compared with the Pearson chi-square test. Before comparing the quantitative variables among different groups, the normality of the variables was verified using the Shapiro-Wilk test. Normally distributed continuous variables were expressed as the mean (standard deviation), while nonnormally distributed variables were recorded as the median (first and third quartiles). The *t*-test and the Mann–Whitney *U* test were used for comparisons between the groups. General linear models (GLMs) correcting for the effects of baseline measures of age, sex, GAT, AL, baseline MD and time on PGs were used to compare DCRs between groups. Risk factors for glaucoma progression were evaluated by logistic regression, and a receiver operating characteristic curve (ROC) was drawn to analyze the area under the curve (AUC). AUC, specificity, and sensitivity were used to reflect the ability of DCRs and risk factors to predict glaucoma progression. A value of *p* < 0.05 was considered statistically significant.

## Results

Seventy-three patients were initially enrolled in the NTG group, of whom 9 patients (9/73, 12%) were lost to follow-up. Among the 64 subjects completing the follow-up, 7 patients (7/64, 11%) did not reach the target IOP. In 57 patients who finished the 4-year follow-up and reached the target IOP, visual fields of 17 patients (17/57, 30%) were diagnosed with progression and 40 patients without progression. In the HTG group, 75 patients were initially enrolled, with 11 patients (11/75, 15%) lost to follow up in the process. Ten of 64 patients finishing the follow-up (10/64, 16%) did not reach the target IOP. In 54 patients who finished the follow-up and reached the target IOP, 20 patients (20/54, 37%) were diagnosed with progression, and 34 patients were without progression.

Baseline characteristics for the nonprogressive and progressive groups of NTG and HTG are detailed in Table 1. There were no significant differences in gender, AL, GAT, CCT, MD or duration of PG use (months) between the two groups in either NTG or HTG. Among HTG patients, the nonprogressive group was younger (*p* = 0.034).

**TABLE 1:** Baseline characteristics of the nonprogressive and progressive groups among NTG and HTG patients.

	NTG			HTG		
	Nonprogressive (N = 40)	Progressive (N = 17)	P	Nonprogressive (N = 34)	Progressive (N = 20)	P
Age [y]	59.5 (14.9)	66.6 (10.6)	0.092	60.0 (11.21)	65.5 (10.4)	**0.034**
Sex [M/F]	25/15	8/9	0.280	24/13	10/10	0.568
AL [mm]	24.58 (1.53)	24.41 (2.20)	0.621	24.17 (23.45, 25.48)	23.95 (23.28, 24.92)	0.243
GAT [mmHg]	13.0 (12.0, 15.0)	13.0 (11.5, 15.5)	0.846	16.0 (14.0, 18.0)	14.5 (13.25, 17.0)	0.291
CCT [µm]	521.4 (30.1)	528.9 (40.7)	0.578	540.5 (33.5)	519.2 (30.2)	0.340
MD [dB]	−5.57 (−8.23, −2.57)	−10.33 (−13.36, −5.09)	0.254	−4.88 (−10.32, −1.70)	−6.76 (−17.32, −3.81)	0.179
PGs [m]	16.0 (0, 73)	36.0 (0, 72.5)	0.978	36.0 (30.9)	31.6 (19.3)	0.664

Data are presented as the mean (standard deviation) or median (Q25, Q75). NTG: normal-tension glaucoma; HTG: hypertension glaucoma; N: number; y: year; M: male; F: female; AL: axial length; GAT: intraocular pressure measured by Goldmann applanation tonometry; CCT: central cornea thickness; MD: mean deviation of the visual field; PGs: prostaglandin duration; m: month. The bold value means p < 0.05.

The comparison of DCRs between the nonprogressive group and the progressive group in NTG is shown in Table 2. In a single variate comparison, there were differences in three parameters at the first applanation (A1), the horizontal length of deflection (A1Length: *p* = 0.013), vertical amplitude of deflection (A1Amp: *p* = 0.010) and deflection area (A1Area: *p* = 0.003), indicating that the NTG progression group had a longer flattening length and larger deformation depth and area.

**TABLE 2:** Comparisons of corneal biomechanics of NTG with and without progression.

		Nonprogressive group (N = 37)	Progressive group (N = 20)	Univariate *p*-value	Multivariate *p*-value
Horizontal length	A1Length [mm]	2.41 (2.31, 2.56)	2.54 (2.42, 2.64)	**0.013**	0.109
	A2Length [mm]	2.97 (2.59, 3.71)	3.89 (2.76, 5.72)	0.056	
Vertical depth	A1Amp [mm]	0.10 (0.10, 0.11)	0.11 (0.10, 0.11)	**0.010**	0.060
	A2Amp [mm]	0.12 (0.11, 0.13)	0.12 (0.10, 0.13)	0.720	
Area	A1Area [mm^2^]	0.19 (0.17, 0.20)	0.21 (0.20, 0.22)	**0.003**	**0.046**
	A2Area [mm^2^]	0.26 (0.20, 0.28)	0.25 (0.21, 0.28)	0.619	
Time	A1T [ms]	7.40 (0.40)	7.26 (0.32)	0.495	
	A2T [ms]	21.87 (21.43, 22.18)	21.80 (21.56, 22.14)	0.565	

Data are presented as the mean (standard deviation) or median (Q25, Q75). NTG: normal-tension glaucoma; A1: the first applanation; Length: deflection length; A2: the second applanation; AMP: deflection amplitude; Area: deflection area; T: time. The bold value means p < 0.05.

After GLM adjustment for age, sex, GAT, CCT, AL, MD, and PG durations, there was still a significant difference between the two groups in the deflection area (A1Area: *p* = 0.046), indicating that the NTG progression group had greater corneal deformation, which further confirmed that the NTG progression group had a more deformable cornea.

**FIGURE 1:**
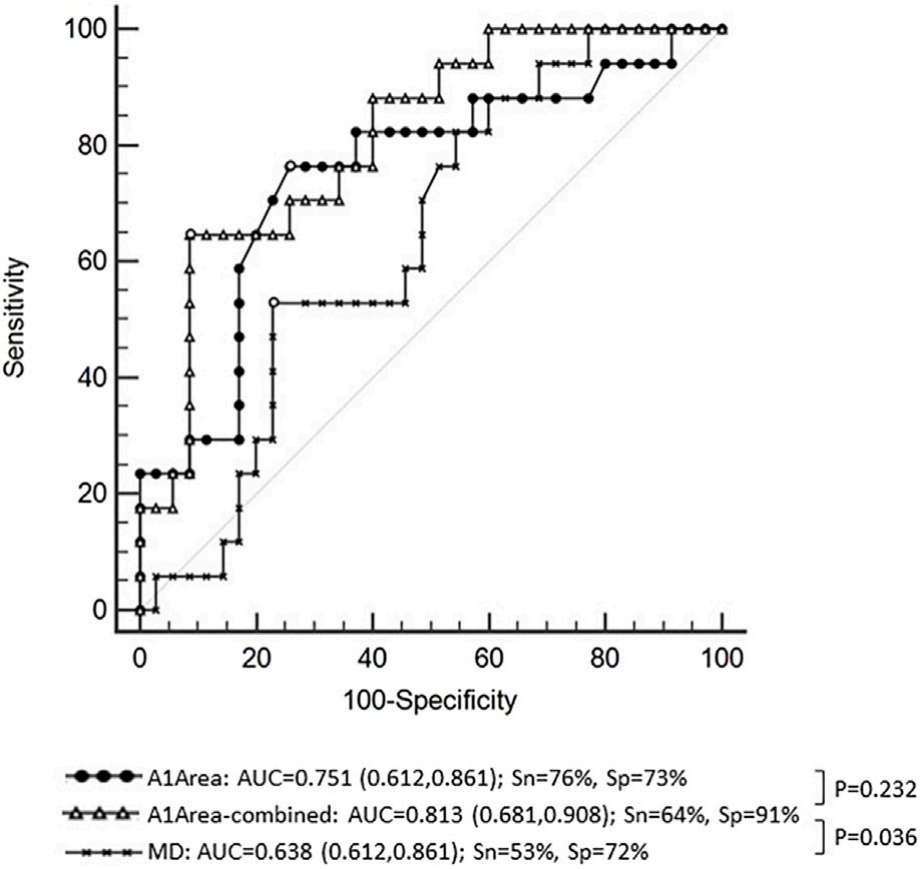
The receiver operating characteristic (ROC) curve of A1Area (deflection area at the first applanation) combined with affecting factors with an area under the curve (AUC) of 0.813 and a sensitivity and specificity of 64% and 91%, respectively. The ROC curve of A1Area-combined factors was similar to that of A1Area, with an AUC of 0.751 (*p* = 0.232), and higher than that of MD (mean deviation of visual field), with an AUC of 0.638 (*p* = 0.036). Sn: sensitivity; Sp: specificity.

We further analyzed risk factors for NTG progression by the logistic regression model. According to the above results, there was a significant difference in A1Area between the progressive group and the nonprogressive group of NTG in the GLM analysis. A1Area and several affecting factors, including age, GAT, CCT, AL, and MD, were included in the logistic regression model. The regression model results are shown in Table 3 and indicated that A1Area was an independent risk factor for NTG VF progression, with an odds ratio (OR) of 1.56/0.1 mm^2^. For every 0.1 mm^2^ increase in A1Area, the risk of NTG progression increased by 1.56 times. The ROC curve of A1Area combined with the above factors to diagnose NTG progression was obtained (Figure 1), with an AUC of 0.813 (95% confidence interval (CI): 0.681, 0.908), sensitivity of 64% and specificity of 91%. When the ROC curve of the diagnosis of NTG progression was plotted with A1Area and MD alone, the AUCs were 0.751 (0.612, 0.861) and 0.638 (0.612, 0.861), respectively (Figure 1). To evaluate the diagnostic performance of A1Area and MD for NTG VF progression, the individual ROC curves for MD and A1Area were compared to the A1Area-combined diagnostic ROC curve. There was no difference between the A1Area and A1Area-combined ROC curves (*p* = 0.232), while the difference between the MD and A1Area-combined ROC curves was significant (*p* = 0.036). The cutoff value of A1Area was 0.198 mm^2^, with a sensitivity and specificity of 76% and 73%, respectively.

**TABLE 3:** Logistic regression model of NTG progression.

	OR	P	95% CI
A1Area	1.56	0.021	1.07, 2.27
age	1.04	0.252	0.97, 1.12
CCT	1.01	0.488	0.99, 1.03
GAT	0.82	0.163	0.61, 1.09
MD	0.93	0.271	0.81, 1.06
AL	1.02	0.932	0.67, 1.56
*R* ^2^ = 0.24, *p* = 0.015			

NTG: normal-tension glaucoma; OR: odds ratio; CI: confidence interval; A1Area: deflection length at the first applanation; CCT: central cornea thickness; GAT: intraocular pressure measured by Goldmann applanation tonometry; MD: mean deviation; AL: axial length.

For HTG, since there was a significant difference in age between the two groups, the DCRs of the two groups were compared by GLM with the affecting factors (age, sex, AL, CCT, MD, and PG durations) adjusted. The results are shown in Table 4. There was no significant difference in DCRs between the two groups.

**TABLE 4:** Comparisons of corneal biomechanics of HTG with and without progression.

		Nonprogressive group (N = 34)	Progressive (N = 20) group	Multivariate *p*-value
Horizontal length	A1Length [mm]	2.47 (2.34, 2.58)	2.42 (2.35, 2.55)	0.148
	A2Length [mm]	2.86 (2.39, 3.59)	3.02 (2.42, 5.63)	0.728
Vertical depth	A1Amp [mm]	0.11 (0.01)	0.11 (0.01)	0.281
	A2Amp [mm]	0.11 (0.11, 0.11)	0.11 (0.10, 0.11)	0.228
Area	A1Area [mm^2^]	0.20 (0.04)	0.19 (0.02)	0.256
	A2Area [mm^**2**^]	0.26 (0.21, 0.29)	0.24 (0.22, 0.30)	0.224
Time	A1T [ms]	7.64 (0.42)	7.71 (0.57)	0.570
	A2T [ms]	21.66 (0.43)	21.45 (0.91)	0.432

Data are presented as the mean (standard deviation) or median (Q25, Q75). HTG: hypertensive glaucoma; A1: first applanation; Length: deflection length; A2: second applanation; AMP: deflection amplitude; Area: deflection area; T: time.

## Discussion

In this study, we found that in NTG subjects, the progressive group had greater A1Amp, A1Area, and A1Length than the nonprogressive group in univariate analysis. The results indicated that the progressive group had a greater corneal depression depth, displacement area and flattening length at the first applanation than the nonprogressive group, which might indicate that the progressive group had a more deformable cornea. As previous studies (Li et al., 2017; Wei et al., 2021; Wu et al., 2022) indicated that cornea biomechanics were related with age, sex, CCT, AL, IOP, and glaucoma severity, these factors were adjusted in the analysis. In baseline comparisons, MD in the progressive group (−10.33 dB) was lower than that of the nonprogressive group (MD: −5.57 dB), while the difference was not significant (*p* = 0.254). Although there were no differences in baseline measures between the two NTG groups, we adjusted them in the study. After adjusting the main factors that may affect corneal biomechanics (age, sex, CCT, AL, GAT, MD), the progressive group still had a larger A1area, indicating that the cornea had a larger deflection area at the first applanation. That is, the corneas of the progressive group were more deformable. Previous studies have shown that the cornea, sclera, and lamina cribrosa are continuous collagenous fibrous tissues and are composed of similar extracellular collagen components (Bellezza et al., 2003; Pakravan et al., 2007). Therefore, the deformability of the cornea may reflect the deformability of the posterior sclera and lamina cribrosa to some extent (Susanna et al., 2019). Sayah et al. (2020) found that more deformable eyeballs were more likely to have glaucoma-related optic nerve damage, including thinning of the ganglion cell complex and retinal nerve fiber layer. In this study, we found that the more deformable the cornea, the more visual field progression in NTG. Therefore, we hypothesized that in the eyeballs of NTG patients, whose cornea is more easily deformed, the cribriform plate tissue may also be easily deformed and less resistant to pressure, leading to optic nerve damage and VF progression being more likely to occur.

We found that for HTG, there was no significant difference in DCRs between the groups with or without visual field progression. The results were consistent with previous observations that corneal biomechanical parameters were not significantly different between the HTG and normal groups (Wei et al., 2021). Our results indicated that VF progression in the HTG group with a 30% decrease in IOP compared with the baseline IOP was not related to corneal biomechanics. In HTG, high IOP may be more responsible for GON, and the specific mechanisms need to be further studied.

To the best of our knowledge, this study is the first one separately assessing corneal deformability of NTG and HTG with or without VF progression. Most previous studies did not separate HTG from NTG in POAG. The study was followed up for 4 years. The results of the study were roughly consistent with those of Li et al. (2018) and Jung et al. (2019). A previous study by our group (Li et al., 2018) found that in NTG, the progressive group had a smaller corneal A1T and a larger DA. Jung et al. (2019) found that corneal DA was greater in POAG progression eyes without separating NTG and HTG. Another study that did not distinguish NTG from HTG obtained opposite results in that thin and hard corneas were more likely to progress (Qassim et al., 2021). Considering the differences in pathogenesis between NTG and HTG (Wei et al., 2021), the classification of NTG and HTG in the analysis of POAG corneal biomechanics is vital. Besides, the parameters used in this study were different and acquired with the whole eye retreat movement eliminated (Wu et al., 2022), which made the results more accurate.

Most scholars believe that glaucomatous optic nerve damage is the result of multiple factors (De Moraes et al., 2011). However, there is still controversy regarding which factors other than IOP are the main risk factors for glaucoma VF progression. Previous studies have found that some factors, such as ocular hypoperfusion (Leske et al., 2004; Dascalu et al., 2021; Fraenkl et al., 2022) and parapapillary vascular factors (Rong et al., 2019; Xu et al., 2022), could affect VF progression. This study suggested that differences in corneal biomechanics may also be one of the reasons for VF progression in NTG patients. The other pathogeneses of NTG remain to be further studied.

Furthermore, this study also analyzed the risk factors for NTG progression and found that the A1Area may be an independent risk factor for NTG progression. The risk of NTG progression increased by 1.56 times for every 0.01 mm^2^ increase in A1Area. The area under the ROC curve of A1Area combined with affecting factors for NTG progression was 0.813 [95% CI: (0.681, 0.908)], with a barely satisfactory sensitivity of 64% and a nice specificity of 91%. When A1Area was used to diagnose NTG progression alone, the AUC was 0.751 (0.612, 0.861), with a reasonable sensitivity of 76% and specificity of 73%. Because visual field MD is an important application in the clinical evaluation of VF progression in patients, the diagnostic value of MD was also analyzed. The area under the ROC curve was only 0.638 (0.612, 0.861). We further compared the diagnostic efficacy of A1Area-combined factors with A1Area or MD alone. The results showed that the diagnostic performance of A1Area was similar to that of A1Area-combined factors, while MD had a significantly lower AUC than A1Area-combined factors. The results revealed that A1area was valuable in predicting NTG VF progression, with a greater increase in A1Area indicating a greater likelihood of progression. This suggests that more attention should be paid to the A1Area increase in clinical practice.

This study has some limitations. First, the small sample size of this study may cause bias in the judgment of corneal biomechanical properties. It is necessary to further expand the sample size to fully assess the role of corneal biomechanics in the progression of glaucomatous fields. Second, previous studies have found that PG drugs may have some effects on corneal thickness and corneal biomechanics in glaucoma patients, but there are controversies. Wu et al. (2016) showed that POAG patients who have used PGs for a long time (more than 2 years) were likely to have more corneal deformation than newly diagnosed patients. Another study from the same group (Wu et al., 2020) found that after a mean follow-up of 10.3 months, the corneal deformation ability of glaucoma patients using PGs decreased. Due to the lack of sufficient literature support for the effects of other anti-glaucoma medications on corneal biomechanics, other drugs were not included in the analysis in this paper. In our study, although there was no significant difference in the duration of PGs use between the two groups, the results of this study still need to be treated with caution. In the next step, more newly diagnosed NTG patients will be included to further evaluate the corneal biomechanical properties. In addition, although CST measurement is based on cornea dynamic reflection of the whole process, it is limited by a lack of direct evaluation of corneal strength based on the tissue. Some novel tools provide information about corneal biomechanical properties, such as the Brillouin optical microscopy (Esporcatte et al., 2020). A more comprehensive assessment of corneal biomechanics will be performed with more devices in the future.

## Conclusion

NTG patients with VF progression had more deformable corneas than those without progression. It was hypothesized that NTG patients with more deformable corneas may also have more deformable cribriform plate tissue, which could accelerate optic nerve damage and VF progression. The A1Area might be an independent risk factor for NTG progression. Further attention should be paid to A1Area in glaucoma patients’ follow-up. There were no significant differences in DCRs between the progressive and nonprogressive groups in HTG. The specific mechanisms underlying this observation need to be further studied.

## Data Availability

The datasets generated and analyzed during the current study are available from the corresponding author on reasonable request.
